# Farm biosecurity measures to prevent hepatitis E virus infection in finishing pigs on endemically infected pig farms

**DOI:** 10.1016/j.onehlt.2023.100570

**Published:** 2023-05-27

**Authors:** Marina Meester, Tijs J. Tobias, Jan van den Broek, Carmijn B. Meulenbroek, Martijn Bouwknegt, Wim H.M. van der Poel, Arjan Stegeman

**Affiliations:** a Farm Animal Health Unit, Department of Population Health Sciences, Faculty of Veterinary Medicine, Utrecht University, Utrecht, the Netherlands; b Royal GD, Deventer, the Netherlands; c Vion Food Group, Boxtel, the Netherlands; d Wageningen Bioveterinary Research, Lelystad, the Netherlands

**Keywords:** HEV, Zoonosis, Foodborne, Within-farm transmission, Risk factors, Mitigation

## Abstract

Hepatitis E virus (HEV) can be transmitted from pigs to humans and cause liver inflammation. Pigs are a major reservoir of HEV and most slaughter pigs show evidence of infection by presence of antibodies (ELISA) or viral RNA (PCR). Reducing the number of HEV infected pigs at slaughter would likely reduce human exposure, yet how this can be achieved, is unknown. We aimed to identify and quantify the effect of biosecurity measures to deliver HEV negative batches of pigs to slaughter.

A case-control study was performed with Dutch pig farms selected based on results of multiple slaughter batches. Case farms delivered at least one PCR and ELISA negative batch to slaughter (PCR^−^ELISA^−^), indicating absence of HEV infection, and control farms had the highest proportion of PCR and/or ELISA positive batches (PCR^+^ELISA^+^), indicating high within-farm transmission. Data about biosecurity and housing were collected via a questionnaire and an audit. Variables were selected by regularization (LASSO regression) and ranked, based the frequency of variable selection. The odds ratios (OR) for the relation between case-control status and the highest ranked variables were determined via grouped logistic regression.

Thirty-five case farms, with 10 to 60% PCR^−^ELISA^−^ batches, and 38 control farms with on average 40% PCR^+^ELISA^+^ batches, were included. Rubber and steel floor material in fattening pens had the highest ranking and increased the odds of a PCR^−^ELISA^−^ batch by 5.87 (95%CI 3.03–11.6) and 7.13 (95%CI 3.05–16.9) respectively. Cleaning pig driving boards weekly (OR 1.99 (95%CI 1.07–3.80)), and fly control with predatory flies (OR 4.52 (95%CI 1.59–13.5)) were protective, whereas a long fattening period was a risk. This study shows that cleaning and cleanability of floors and fomites and adequate fly control are measures to consider for HEV control in infected farms. Yet, intervention studies are needed to confirm the robustness of these outcomes.

## Highlights

• On some HEV infected farms, groups of pigs can be raised HEV free for slaughter.
• Measures associated with delivery of HEV free groups of pigs to slaughter were found.
• More frequent and effective cleaning of pig pens and fomites may reduce spread of HEV.
• Fly control may prevent spread of HEV between different groups of pigs within farms.
• Reducing HEV spread within farms will likely reduce HEV exposure of pork consumers.

## Graphical abstract

**Figure:**
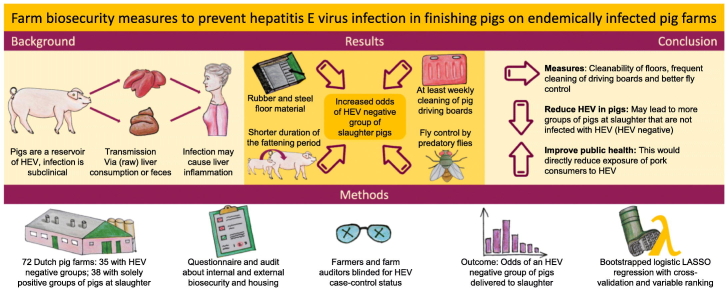

## 1 Introduction

Yearly, an estimated 20 million human hepatitis E virus (HEV) infections occur worldwide [1]. HEV is a single-stranded quasi-enveloped RNA virus [2] and infection may lead to acute liver inflammation, acute liver failure or chronic liver cirrhosis [3] and extrahepatic manifestations like Guillain-Barré syndrome [4]. In Europe (EU), HEV genotype 3 is most common and HEV seroprevalence in blood donors from EU countries ranges from 1.1% (Spain) to 52% (France) [5]. It appears that the major pathway for HEV genotype 3 infections in humans is foodborne transmission, by raw or undercooked pork, liver sausages in particular [6].

Pigs are a major reservoir of HEV. The seroprevalence of HEV in pigs has been reported for many EU countries and ranges from 8 to 93%, and average seroprevalence in Dutch pig farms exceeds 70% [7]. In general, pigs are infected orally and shed the virus in feces and probably urine [8]. Moreover, HEV may persist in livers of pigs, with 11% of livers testing PCR positive in slaughter aged pigs [9]. The zoonotic nature of the virus and wide dissemination of HEV in pig farms signify the need to understand how HEV transmission in pig farms can be controlled and delivery of HEV infected pigs to slaughter prevented, in order to improve public health.

Limited knowledge is available on measures that could aid control of HEV in endemically infected pig farms. A few case-control studies that compare management and biosecurity on pig farms with high versus low or absent (sero)prevalence of HEV have been performed in the EU and countries with a similar pig farming system [[10], [11], [12], [13]]. Mainly external biosecurity measures like quarantining pigs that enter the farm [11,13], enforcing showering [12] and specific boots [10] before farm entrance, and using a public instead of a private water source [10], are associated with lower HEV seroprevalence or prevalence in pig farms.

Still a lower seroprevalence does not imply a lower risk for the consumer, as pigs may be infected shortly before slaughter and not have seroconverted by that time. Nor does a low proportion of PCR positive pigs at slaughter indicate a low prevalence of HEV infected pigs in farms, as HEV infection may have occurred earlier in that group of pigs and been cleared already. Testing slaughter pigs for the presence of virus and antibodies provides information for both recent and earlier infection. Pigs from HEV infected farms that arrive at the slaughterhouse in which neither virus, nor antibodies are detected, point at the presence of housing and/or internal biosecurity measures that reduce within-farm HEV transmission. Therefore, the aim of the current study is to identify biosecurity measures and housing characteristics associated with delivering batches of pigs to slaughter that test negative for HEV infection both by PCR and ELISA.

## 2 Materials and methods

### 2.1 Study design and sampling

Pig farms in the Netherlands were visited for an audit and interview with the farmer, about housing and biosecurity on the farm, to perform a case-control study. The pig farms were selected based on results from a prevalence study. A full description of the sampling strategy, inclusion criteria and laboratory test details in that study is available elsewhere [7]. In short, 215 pig farms that delivered pigs to three Dutch abattoirs were selected. Selection was done randomly for organic and conventional pig farms. Organic farms produce pigs according to the European Commission Regulation (EC889/2008), including the obligation for outdoor access for pigs. Repeated cross-sectional sampling of pigs from batches delivered to slaughter was performed and a median of six blood samples per batch was collected, between January and August 2019. A batch is defined as all pigs originating from one unique farm that are delivered to slaughter simultaneously. Therefore, a batch can resemble a group of pigs housed together on the farm. On average eight different batches per farm were included. Blood samples were analysed individually for HEV antibodies by an ELISA, and they were pooled per batch to test for HEV RNA by reverse transcription polymerase chain reaction (RT-PCR). Samples were stored at −20 °C until analysis.

### 2.2 Farm selection criteria

The 215 pig farms in the prevalence study had an average seroprevalence of 76%. All farms delivered at least one seropositive pig to slaughter. Although no HEV negative farms were identified, ELISA and PCR results per batch could be used for a cluster analysis, that retrieved four farm clusters with varying within-farm transmission patterns [7]. One cluster (number 4) consisted of farms that were able to deliver at least one batch to slaughter that was both PCR negative and with at least five out of six pigs seronegative (PCR^−^ELISA^−^ batch) [7]. It is hypothesized that the presence of PCR^−^ELISA^−^ batches points at low within-farm HEV transmission and that its occurrence is associated with farm housing and biosecurity measures.

**Fig. 1:**
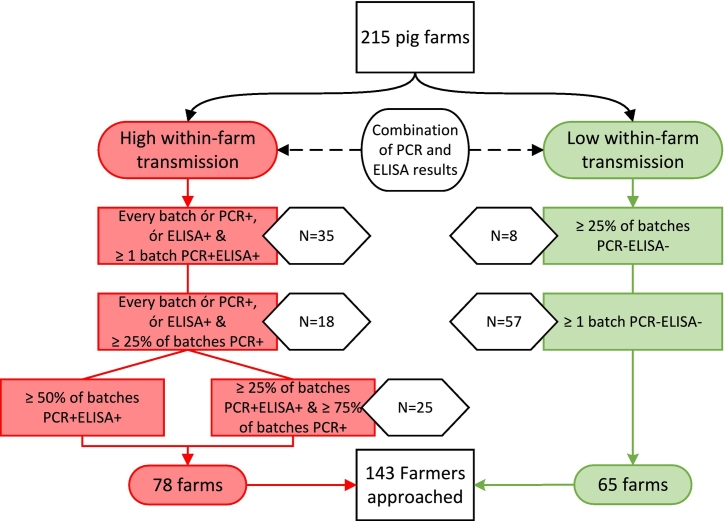
Selection criteria for high vs. low within-farm transmission farms and number of farms approached per step. Legend: Red boxes (): Farms with high transmission of HEV, i.e. a higher amount of batches that are either or both PCR or ELISA positive. Green boxes (): Farms with low transmission of HEV, i.e. at least 1 batch that is PCR and ELISA negative (PCR^−^ELISA^−^). The criteria for selecting farms had to be loosened twice because of insufficient willingness of farmers to participate, which is represented by the different green and red rectangle boxes below each other and number (N) of farms approached per selection criterion are shown in white hexagons (). (For interpretation of the references to colour in this figure legend, the reader is referred to the web version of this article.)

On that account, for the current case-control study on the one hand farms with the most PCR^−^ELISA^−^ batches were selected (Fig. 1., green boxes). On the other hand the farms with the most PCR positive as well as ELISA positive batches were selected, as those presumably have the highest within-farm transmission of HEV (Fig. 1., red boxes). In total, 143 farms were eligible and approached for participation in this case-control study.

### 2.3 Questionnaire and audit design

During farm selection no information was available on farm characteristics. To investigate measures related to low HEV within-farm transmission, a questionnaire (Q) and audit (A) were designed. All risk factors for HEV farm prevalence published in previous studies were included in the Q and A [[10], [11], [12]]. The specific questions per topic were discussed with an independent veterinary specialist in porcine health management (dipl. ECPHM) (Table 1). Also, the Q and A were pretested at three farms, to check feasibility, understanding and interpretation of questions by the farmers.

**Table 1:** Main themes, subthemes, topics and number (N) of questions in the questionnaire (Q) and audit (A).

Theme	Subtheme	Questions	Q (N)	A (N)
General farm characteristics				
Farm type		Organic or conventional; production stages; Own breeding gilts; genetics	16	
Personnel		Number; function; For specific tasks	15	
Hepatitis E virus		Knowledge; Importance	2	

Animals, size, production parameters				
Buildings		Specific for production stage; Age; Number	4	
Farm size		Number of accommodations per production stage; Number of batches per year	8	
Diseases and vaccinations		Salmonella; PRRSv; Influenza; Defined daily dose; Vaccinations per production stage	37	
Production weaners		Mortality; Age at weaning; Weight	6	1
Production fatteners		Mortality; Age at fattening; average daily gain; feed conversion rate; difference in age between pigs at slaughter	7	1

Feed, water, manure				
Feed		Feed type; system; acidification; feed remainders	20	2
Water origin		private source or municipality; age of private source	5	
Water cleaning		additional substances in water; cleaning of water system and water	7	
Manure		Frequency of emptying manure pit; frequency of pen befouling, closed floors	11	24

External biosecurity				
Hygiene lock		Shower; clothing; boots; contact other pig farms	12	21
Loading and unloading place		Same place for loading and unloading; cleaning; walking route passes loading place	8	9
Quarantine		Presence; usage; separate manure pit and air supply	4	7

Internal biosecurity				
Other animal species		Other farm animals; pets; pigs of other farms	18	3
Pest control		Presence; protocol; company or private; successfulness; method for control of flies	11	7
Cleaning		Frequency of cleaning pens; corridors; ceilings; boots; clothes; boards; Method for cleaning pens; corridors; boots	69	
Disinfection		Frequency of disinfecting pens; corridors; boots; clothes; boards; Type of disinfectants; time between cleaning and disinfection	26	
Cleanliness of materials		Overalls; boots; corridors; pens; outdoor pens; General score for cleanliness inside and outside farm; floor material		47

Direct contact between pigs				
Farrowing		cross-fostering; all in - all out (AIAO)	18	8
Weaning		mingling during weaning; pen density; transferring pigs to weaning compartment; AIAO	17	24
Fattening		mingling during fattening; pen density; AIAO	19	22
Sick-bay		Presence; Emptiness; return from sick-bay to other compartments; specific compartment	6	22

Indirect contact between pigs				
Between farm compartments		Treatment round; gloves; walking routes; hygiene lock per production stage	17	36
Within farm compartments		Period of emptiness compartment; showering of sows; gilt acclimatization	6	4
Farm equipment, materials, carcasses		Needles; enrichment; equipment per production stage; carcass removal and storage	19	

The final Q was developed in Microsoft Access [14] and existed of 388 questions, of which 210 were binary (true or false), 98 categorical, 33 continuous and 47 open. The A consisted of 238 questions/checkpoints, of which 122 were binary, 43 categorical, 50 continuous and six open.

### 2.4 Farm visits

Farm visits were done by eight people (the first author and seven students of veterinary medicine and applied animal sciences) in duos, between March and October 2020. The first author trained the students by jointly practicing the Q and A at three farms that were not included in the study. The composition of the duos alternated to limit observer bias. Farmers and farm auditors in the project were blinded for HEV farm prevalence.

### 2.5 Data cleaning

The Q and A responses were manually checked in MS Excel™ for obvious typographic errors [15]. Besides, ‘other answer, namely’-variables were categorized and combined with the categorical question they belonged to. In R [16], variables were checked for variation in answers and having sufficient answers per answer category. Continuous variables (*n* = 83) were categorized, because the association with the outcome does not necessarily have to be linear. Questions with less than five answers per category were recategorized by combining categories.

### 2.6 Missing and associated data

Variables that were asked both in the Q and A or that were very similar, were assessed for association by χ^2^ test, or Fisher's exact test in case the χ^2^ assumptions were not met, to reduce the number of noise variables in the model. In case the χ^2^ *p*-value was below 0.05, one of two variables was taken out of the dataset. Variables with >15% missing values (arbitrary cut-off) were excluded from further analysis. Multiple imputation by chained equations (MICE) was performed to prevent loss of power in the multivariable analysis [[17], [18], [19]]. Using this method five multiple imputed datasets (MIDS) were retrieved for subsequent statistical analysis.

### 2.7 Statistical analysis

Although farms were selected as being a case (delivered PCR^−^ELISA^−^ batches) or a control (did not deliver PCR^−^ELISA^−^ batches) based on assumptions of low or high within-farm transmission, a binomial outcome would oversimplify the outcome as there is information available on the number of PCR^−^ELISA^−^ and positive batches. Therefore, grouped logistic regression was performed, which resulted in odds ratio (OR) estimates for having a PCR^−^ELISA^−^ batch. The association between all potential factors and the outcome was assessed by least absolute shrinkage and selection operator (LASSO) regression with the CRAN package glmnet [20]. LASSO regression is a multivariable regularization model that provides sparser models than traditional regression models and is able to handle multicollinearity [21,22]. The regularization leads coefficients to have reduced absolute values and some to be shrunken to zero. Regularization is controlled by a metaparameter λ. A variable is selected when the absolute value of its correlation with the outcome is larger than λ [23]. The value of λ (optimal value) is determined by selecting the value with minimum mean cross-validated prediction error in 10-fold cross-validation [22].

Despite applying cross-validation, the optimal value of λ can vary between model runs. Also, especially in case of small effect sizes, LASSO may select false positive variables according to several simulation studies [24,25]. Therefore, a stability analysis was performed, by nesting cross-validation for λ selection within bootstrap sampling (500 times). The latter step was used to make the variable selection sparser and more precise in two ways: variables with bootstrap confidence intervals (CIs) of coefficients that contain zero could be excluded [24]; A ranking of stability could be made based on the number of times variables were selected in 500 bootstrapped LASSO models (i.e. the number of times each variable had a coefficient larger than zero) [26].

The results of the bootstrapped LASSO models were compared between the five MIDS. Finally, a multivariable grouped logistic regression model was used to determine the ORs for having an HEV PCR^−^ELISA^−^ batch at slaughter, for the most stable variables.

## 3 Results

### 3.1 Farms

**Fig. 2:**
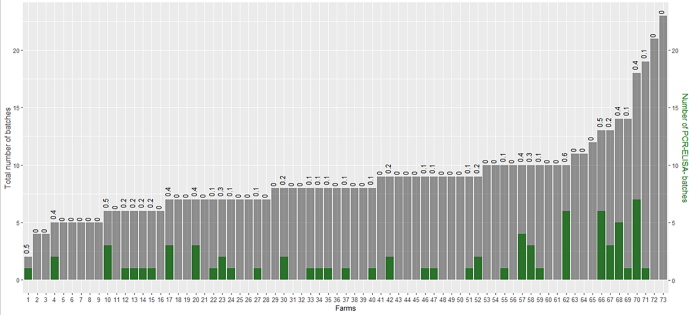
Barplot of the number of PCR^−^ELISA^−^ batches per farm, compared to the number of total batches sampled. Legend: Total number of batches are shown in grey, and number of PCR^−^ELISA^−^ batches in green. Results are ordered from lowest to highest number of batches sampled per farm. Bar labels give the proportion of batches sampled that is PCR^−^ELISA^−^. (For interpretation of the references to colour in this figure legend, the reader is referred to the web version of this article.)

Of 143 approached farmers, 73 were willing to participate (overall participation rate 51%), consisting of 35 farms with at least one PCR^−^ELISA^−^ batch and 38 without any PCR^−^ELISA^−^ batches. The number of sampled batches per farm ranged from two to 23 and the proportion of PCR^−^ELISA^−^ batches from 0.1 to 0.6 (Fig. 2). Table 2 shows the baseline results of the low and high within-farm transmission farms in the study and the average results for all farms in the previous prevalence study [7].

**Table 2:** Serological and PCR results of included farms with and without PCR^−^ELISA^−^ batches and all 215 farms [7].

	Farms with at least 1 PCR^−^ELISA^−^ batch (cases)	Farms without PCR^−^ELISA^−^ batches (controls)	Average results of 215 farms [7]
Average farm seroprevalence, i.e. proportion of seropositive pigs per farm (IQR)	57.0% (39.0–72.6%)	84.3% (78.6–90.4%)	73.6% (66.7–87.2%)
Average proportion of PCR positive batches per farm, based on pooled serum per batch (IQR)	27.8% (11.8–44.4%)	47.0% (31.2–69.2%)	40.2% (25.0–57.1%)

### 3.2 Data cleaning

**Fig. 3:**
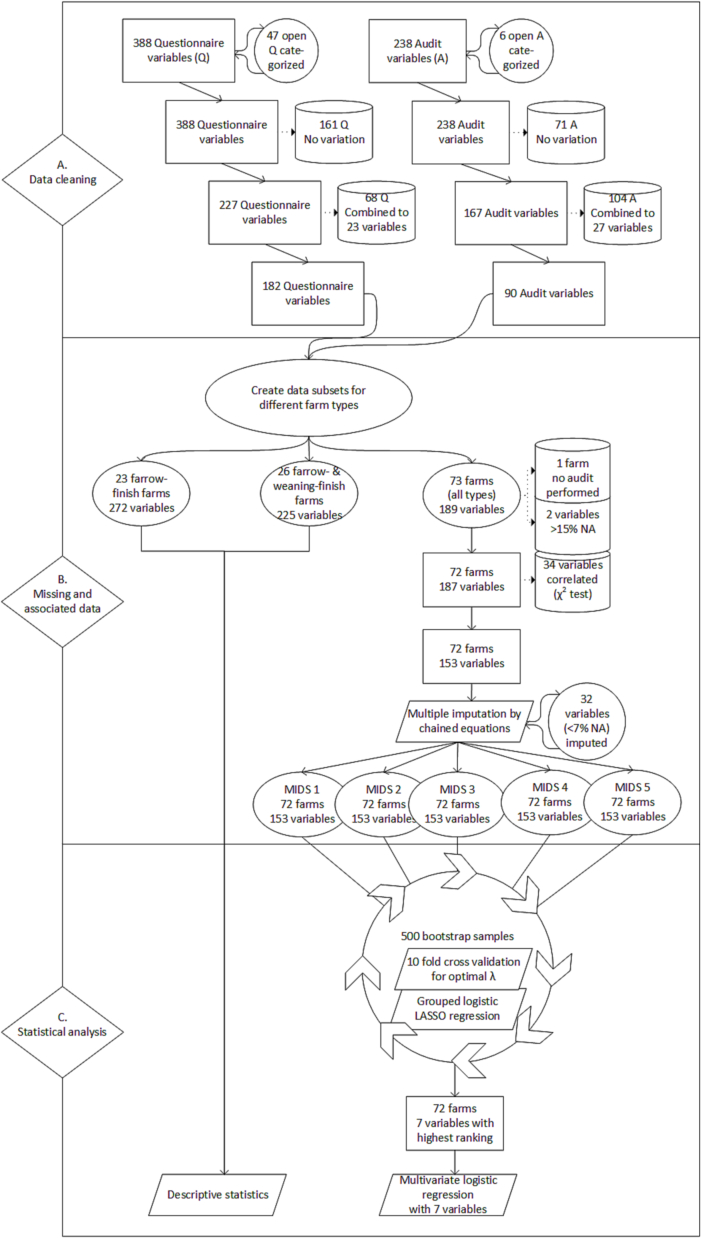
Flowchart of the steps in data processing in three sections; data cleaning, missing and associated data and statistical analysis. Legend: Rectangles () represent the number of variables and farms at a certain step in data processing, while cylinders () represent steps to clean the data and parallelograms () steps in the statistical analysis. Q: questionnaire; A: audit; NA: non-available; MIDS: Multiple imputed dataset.

Fig. 3, section A shows the data cleaning process. For the A, many variables were combined (104 combined to 27 variables) because for both weaning and fattening compartments four different pens were audited and results were averaged over the four pens per production stage. After data cleaning, 128 Q and 90 A variables remained.

### 3.3 Missing and associated data

Because specific questions were asked per production stage, subsets of the dataset were made (Fig. 3, section B). The subsets for farrow- and weaning-to-finish farms did not contain sufficient farms for analysis, but descriptive statistics are provided (Supplementary Table A). For the subset of variables for all farms, one farm was taken out because the A could not be performed there.

Variables were dropped for several reasons (Fig. 3, section B) and 32 variables in the subset (with 1.4 to 6.9% missing values) were imputed to obtain five MIDS.

### 3.4 Statistical analysis

**Fig. 4:**
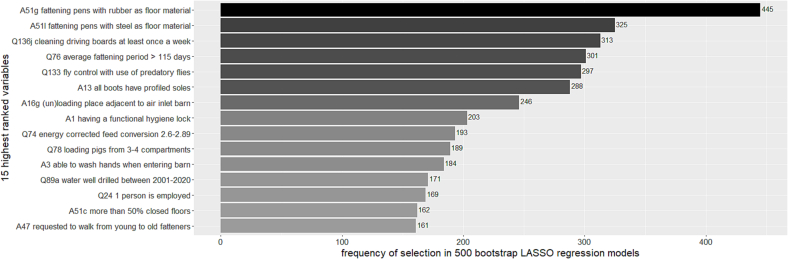
Barplot of how often variables from the questionnaire and audit were selected by 500 bootstrapped LASSO regressions.

The bootstrapped logistic LASSO regression with cross-validated λ selection was performed on a dataset with 153 variables, for all 5 MIDS (Fig. 3, section C). Variables were selected between 0 and 445 times out of 500 bootstrap samples without large differences between the MIDS. Fig. 4 displays how often the 15 highest ranked variables were selected on average. The first seven variables were selected for the final model, because the marginal difference in how often variables were selected is higher between variable 7 (unloading place next to air inlet) and 8 (functional hygiene lock) than between variable 6 (boots have profiled soles) and 7. The bootstrap 95% CI of the coefficients of these 15 variables were all below or above one so the final model could not be made sparser by looking at CIs [24]. Therefore the final selected variables that are positively associated with delivery of PCR^−^ELISA^−^ batches are rubber and steel floor material in fattening pens, weekly cleaning of pig boards (used to move pigs between farm compartments), and fly control, in particular by predatory flies. A fattening period (Last stage before pigs go to slaughter) that lasts longer than 105 days, wearing boots with profiled soles and a loading or unloading place next to the air inlet of a barn are negatively associated with delivery of PCR^−^ELISA^−^ batches. The ORs and 95% CIs of the multivariable logistic regression model can be found in Table 3.

**Table 3:** Odds ratios and 95% confidence intervals in final multivariable logistic regression model, for having an HEV PCR^−^ELISA^−^ batch of pigs delivered to slaughter, with variables in order of frequency of selection by bootstrapped LASSO regression.

Variable	A or Q	Odds Ratio	2.5%	97.5%
Fattening pens without rubber as floor material	A	Reference	–	–
Fattening pens with rubber as floor material	A	5.87*	3.03	11.6
Fattening pens without steel as floor material	A	Reference	–	–
Fattening pens with steel as floor material	A	7.13*	3.05	16.9
Cleaning pig boards never or less than once a week	Q	Reference	–	–
Cleaning pig boards once a week or after every pig contact	Q	1.99*	1.07	3.80
Average fattening period (2019) ≤ 105 days	Q	Reference	–	–
Average fattening period (2019) >105 ≤ 115 days	Q	0.26*	0.12	0.58
Average fattening period (2019) > 115 days	Q	0.21*	0.09	0.45
No fly control	Q	Reference	–	–
Fly control with pesticides sprayed on walls	Q	1.34	0.54	3.34
Fly control with pesticides in manure pit	Q	1.75	0.73	4.35
Fly control with use of predatory flies	Q	4.52*	1.59	13.5
All boots have smooth soles	A	Reference	–	–
Some boots have smooth, others have profiled soles	A	0.65	0.27	1.57
All boots have profiled soles	A	0.81	0.32	2.27
(Un)loading place away from air inlet barn	A	Reference	–	–
(Un)loading place right next to air inlet barn	A	0.80	0.41	1.54

## 4 Discussion

The aim of this study was to identify biosecurity measures and housing characteristics that are associated with the delivery of batches of pigs to slaughter that are PCR negative and seronegative (PCR^−^ELISA^−^). This contributes to the understanding of how to reduce transmission of HEV within endemically infected pig farms and to ultimately reduce the proportion of positive pigs at slaughter and exposure of pork consumers to HEV. Furthermore, hepatitis E virus control on pig farms is essential for a One Health approach to prevent zoonotic HEV infections in humans through reduction in transmission via other routes, such as direct pig contact and environmental transmission.

Rubber and steel on the floor of fattening pens were significantly associated with the odds of a PCR^−^ELISA^−^ batch of pigs with ORs of 5.9 and 7.1 respectively, and had the highest ranking in the bootstrapped grouped logistic LASSO regression. Steel as floor material is often used as slatted floor, for feces and urine to run down into the manure pit. Steel slats are narrower than concrete slats, so having steel may reduce the chance that pigs have contact with HEV contaminated feces of pen mates. Rubber is used for floor areas where the floor may otherwise quickly deteriorate, for instance in front of the feed trough (Dr. P. van der Wolf, dipl. ECPHM, personal communication). Moreover, when rubber or steel is used as part of the pen floor, by definition less floor surface is made of concrete. Concrete is a porous material and consequently more difficult to clean after the pigs in the pen have been sent to slaughter. Therefore, rubber and steel as floor materials may contribute to reduced transmission of HEV between consecutive batches of pigs within compartments.

The frequency of cleaning pig driving boards, the type of measure used to control flies and the type of sole below boots may all contribute to prevention of transmission between groups of pigs that are simultaneously present in the barn. Pig driving boards are used to move pigs between barns and if not cleaned properly and frequently, HEV may be spread mechanically between pigs of different locations within the farm. The cleanability of boots with profiled soles is less than those with smooth soles. As a result feces may be more easily carried from compartment to compartment by using boots with profiled soles, leading to a lower likelihood (insignificant) of an HEV PCR^−^ELISA^−^ batch. Three measures to control flies all increase the odds of delivering PCR^−^ELISA^−^ batches of pigs to slaughter compared to not applying fly control. Flies have been described to be mechanical vectors for several viruses in pig farms, such as porcine circovirus 2b, rotavirus and porcine reproductive and respiratory syndrome virus [[27], [28], [29], [30]]. Besides mechanically spreading pathogens, biting flies could theoretically also spread HEV by consecutively biting viremic and susceptible pigs. The likelihood of these transmission routes via flies, however, have not been elucidated.

A longer fattening period was associated with lower odds for delivering a PCR^−^ELISA^−^ batch of pigs to slaughter. A previous risk factor study showed that a large age gap between the youngest and oldest pig in a batch increases the risk of HEV positive livers at slaughter [10]. Both risk factors correspond to age dependent HEV results reported in other studies, namely that HEV seroprevalence rises with the age of pigs [[31], [32], [33]] and prevalence falls with age [34]. Other risk factors found in previous studies were included in the questionnaire and audit but have not been found to be associated with HEV in the current study. For instance, external biosecurity factors like having a quarantine period, a sanitary ford, or contact between pigs and other domestic species [11] as well as demanding showering and wearing farm-specific boots for visitors before coming into the farm [12] were included in the questionnaire but are not associated with delivering PCR^−^ELISA^−^ batches to slaughter. This was expected, because external biosecurity measures can prevent pathogens from entering farms, though cannot prevent transmission of pathogens within farms that are already infected, which was the case for the current study.

Using an audit besides a questionnaire showed to be advantageous in this study. Firstly, several parameters could only be scored objectively using the audit, such as the number of flies in farms. Moreover, incongruence between responses of farmers and farm auditors could be assessed, for instance to the question whether pets are allowed inside the barn. Lastly, response bias could be reduced as farmers realized that inaccurate (socially desired) responses could come to light during the audit. Instead of a questionnaire specific for this study, a standardized protocol to assess farm biosecurity like the Biocheck.UGent™ tool could have been used [35]. However, such tools provide feedback on the applied biosecurity measures relative to a benchmark, while there is no consensus on relative importance for HEV. Besides, such tools frequently generate weighted total scores for biosecurity, while we aimed for specific measures [36]. The tools could contribute to future control of HEV because farmers and veterinarians can use them to periodically assess the biosecurity level of farms.

To summarize, internal biosecurity measures, such as more efficient cleaning of pig pens, frequent cleaning of fomites like pig boards, and fly control, may reduce transmission of HEV within farms. As pigs are a major reservoir of HEV and nearly all farms are estimated to be HEV infected, reducing the within-farm transmission is the first necessary step to reduce the number of HEV infected pigs at slaughter and thereby reduce exposure of pork consumers to HEV. An intervention study that proves the effectiveness of measures to keep a farm compartment free from HEV, within an endemically infected farm, is necessary to demonstrate inference.

## Ethics statement

The authors confirm that the Ethics Review Board of the faculties of Science and Geosciences of Utrecht University has approved this study, and the ethical policies of the journal, as noted on the journal's author guidelines page, have been adhered to.

## Funding

This work was part of the project “HEVentie: hepatitis E virus intervention in primary pig production”. HEVentie receives financial support of the Topsector AgriFood (TKI AF 18119). Within TKI Agri&Food private partners, research institutes and government cooperate to innovations for safe and healthy food for 9 billion people on a resilient globe. The work was supported by the 10.13039/100010661European Union's Horizon 2020 Research and Innovation Programme under grant agreement No. 773830: One Health European Joint Programme (BIOPIGEE: Biosecurity Practices for Pig Farming Across Europe).

## CRediT authorship contribution statement

**Marina Meester:** Conceptualization, Formal analysis, Visualization, Investigation, Writing – original draft. **Tijs J. Tobias:** Conceptualization, Supervision, Project administration, Writing – review & editing. **Jan van den Broek:** Methodology, Writing – review & editing. **Carmijn B. Meulenbroek:** Formal analysis, Investigation, Writing – review & editing. **Martijn Bouwknegt:** Conceptualization, Supervision, Writing – review & editing. **Wim H.M. van der Poel:** Conceptualization, Supervision, Writing – review & editing. **Arjan Stegeman:** Conceptualization, Supervision, Writing – review & editing.

## Declaration of Competing Interest

The authors declare that they have no competing interests.

## Data Availability

The raw data that support the findings of this study are not publicly available due to privacy restrictions. Data on the number of answers per question in the questionnaire and audit, in relation to having an HEV PCR^−^ELISA^−^ batch is provided in Appendix A, Supplementary Data.

## Appendix A Supplementary data

Supplementary material Image 7
